# Predicting risk of progression of early to late AMD in the aging eye through imaging and multimodal evaluation: PRIME study protocol

**DOI:** 10.1371/journal.pone.0355953

**Published:** 2026-08-31

**Authors:** Ali Forouhari, Chui Ming Gemmy Cheung, Low Lian Leng, Noa Gilead, Usha Chakravarthy, Kelvin Yi Chong Teo

**Affiliations:** 1 Singapore Eye Research Institute, Singapore National Eye Centre, Singapore; 2 Duke-NUS Eye ACP, National University of Singapore, Singapore; 3 Singapore General Hospital, Singapore; 4 Centre for Public Health, Faculty of Medicine and Health Sciences, Queen’s University Belfast, Belfast, United Kingdom; PLOS: Public Library of Science, UNITED STATES OF AMERICA

## Abstract

**Background:**

Age-related macular degeneration (AMD) is a leading cause of vision impairment globally and demonstrates substantial variation regarding progression and clinical subtypes across populations. This paper describes the methodology of our study, designed to establish the natural history of AMD in Asian elderly and to identify predictors of progression from early signs of AMD to late AMD over a 5-year period.

**Methods:**

This prospective, longitudinal cohort study aims to recruit 1500 participants from community-based cohorts in Singapore, with a follow-up duration of 5 years. Eligible participants are adults aged 55 years or older without severe systemic illness that would preclude participation.

At baseline, demographic data and medical history will be collected, and visual acuity will be assessed. A basic imaging protocol including color fundus photography (CFP), optical coherence tomography (OCT), and OCT angiography (OCTA) will be performed. Blood samples will be collected from consenting participants. Images will be graded for early signs of AMD, defined as the presence of pathological findings on CFP, OCT, or OCTA.

Participants without early signs of AMD will undergo annual follow-up using the basic imaging protocol. Those with any pathological findings at baseline or during follow-up will undergo an enhanced imaging protocol every 6 months, including ultrawide-field fundus photography, fundus autofluorescence (FAF), OCT, and OCTA. All follow-up visits will be conducted at the Singapore Eye Research Institute (SERI).

Multimodal imaging biomarkers will be integrated with clinical, demographic, genetic, and metabolomic data to assess the relative risk of progression and identify potential predictors. The study protocol has been approved by the SingHealth Centralised Institutional Review Board.

**Discussion:**

There is a paucity of data on the progression of AMD and optimal referral thresholds, particularly in Asian populations. This community-based cohort, representative of the general population, will generate one of the largest longitudinal datasets on AMD in Asia and aims to inform future screening strategies.

Study registration number: ClinicalTrials.gov Identifier, NCT07653269.

## Introduction

Age-related macular degeneration (AMD) is a leading cause of vision impairment globally, particularly among individuals aged 50 and older, posing a significant challenge to public health [1,2]. AMD primarily affects the macula, leading to progressive central vision loss that severely impacts daily activities such as reading, driving, and recognizing faces, thereby diminishing patients’ overall quality of life [3,4]. With the global population aging rapidly, particularly in regions like Europe, North America, and Asia, the prevalence of AMD is projected to rise sharply [1]. This trend has profound implications for public health systems, demanding enhanced healthcare resources and interventions to manage the growing burden of vision impairment.

Despite its global impact, the incidence, clinical presentation, progression, and subtypes of AMD show significant variation across different populations [5,6]. Large-scale epidemiological studies, such as the Singapore Epidemiology of Eye Diseases (SEED) study and Beijing Eye Study, have shed light on these population-specific differences, highlighting unique AMD phenotypes and risk factors among Asians [6–9]. For instance, polypoidal choroidal vasculopathy (PCV), a distinct subtype of AMD, is notably more prevalent in Asian populations compared to Western populations, where traditional AMD hallmarks, such as drusen or soft drusen, are more commonly observed [9,10]. These distinctive features underscore the necessity for Asian-specific research to better understand the underlying mechanisms, risk factors, and clinical manifestations of AMD in this population.

While there is consensus regarding features that predispose to late AMD, at an individual level, risk, rate, and pattern of progression remain unpredictable; therefore, there is a need to develop effective AMD screening. This contrasts with diabetic retinopathy because the natural history is well understood, and the risk of vision loss increases sharply in those with referable DR. In AMD, this threshold is not yet established.

This paper describes the methodology of our study, designed to track retinal aging changes and understand the natural progression of AMD in a multi-ethnic Asian population. We hypothesize that there are specific high-risk features identifiable through multimodal imaging and systemic biomarkers that are associated with progression of AMD.

## Methods

### Study setting

This is a prospective, longitudinal cohort study designed to recruit participants from community cohorts in Singapore. Community cohorts were selected specifically to ensure the study cohort was generalizable to an overall population. Following the recruitment and baseline visit, over a five-year period, participants will undergo follow-up ophthalmic assessments at the Singapore Eye Research Institute (SERI).

Our specific aim is to establish the natural history of AMD in Asian elderly and to identify clinical multimodal imaging, genetic, and metabolomic predictors of progression. Progression is defined as the change from early signs of AMD to advanced AMD. Our secondary aims include: 1- define thresholds for referable early AMD suitable for community screening, 2-formulate an Asian-specific image classification system against existing AMD frameworks.

This will ultimately lead to developing personalized monitoring strategies for at-risk individuals and also inform a comprehensive and standardized community screening approach for the early detection and monitoring of AMD.

### Sample size

Previous population-based data from the Singapore Epidemiology of Eye Diseases (SEED) study reported an incidence of progression to late AMD of 0.51% among adults aged ≥40 years [10]. However, a higher progression rate is anticipated in the present study, as participants aged ≥55 years will be recruited.

The target sample size of 1,500 participants was selected to investigate progression to late AMD in an Asian community-based cohort. This sample size was determined based on feasibility and anticipated recruitment and 5-year follow-up capacity.

### Participant enrollment

Potential patients will be recruited through SingHealth Community Outreach Programme (or other smaller community-based screening projects led by our study team) and Outram Community Hospital (OCH).

The SingHealth Community Outreach Programme is an ongoing, established comprehensive initiative aimed at improving health outcomes by bringing essential healthcare services directly to the community. The current study will add an eye research component to this population health initiative.

This program, with a multi-site approach based on three main general hospitals in Singapore (Singapore General Hospital (SGH), Changi General Hospital (CGH), and Sengkang General Hospital (SKH)), focuses on accessibility and preventive care, and addresses a wide range of health concerns, including chronic diseases screening, frailty screening, and functional screening, etc. The program ensures underserved populations receive timely medical attention, health education, and referrals to help residents to keep well, get well, and live well. Through these efforts, SingHealth strives to promote healthier communities and reduce the burden of preventable diseases.

Our study adds an eye screening module (specifically for AMD), and this greatly enhances the overall impact of the SingHealth Community Outreach Programme and other community-based screening projects, further improving health outcomes.

The study team will also recruit participants from OCH. Patients at OCH are typically older adults, with an average age of 72 years old, who are usually transferred from acute hospitals such as SGH and national centers for rehabilitation and subacute care. The most common conditions are hip fracture, stroke, and rehabilitation after an acute illness. Their length of stay is an average of 28 days before returning to the community. As 95% of them return to the community, this represents an opportunity as a captive audience to perform AMD screening in this group of older adults. The hospital team and the study team will work together to identify probable participants according to the inclusion criteria:

### Inclusion criteria

- Adults aged 55 years or older
- Willing and able to undergo protocol-required procedures for both eyes
- Willing and able to provide written informed consent

### Exclusion criteria

- Systemic disorders that preclude reliable clinical examination or multimodal imaging
- Poor compliance or severe mental illness that hinders participation
- Persons who are unable to give informed consent

Participants may withdraw from the study at any time without affecting their medical management or statutory rights.

### Ethical consideration

This study will be conducted in accordance with the ethical principles that have their origin in the Declaration of Helsinki and that are consistent with the Good Clinical Practice.

The final study protocol, including the Participant Information and Consent Form, has been approved by the SingHealth Centralised Institutional Review Board (CIRB) (protocol code 2025−0160)

Participants will first be identified and recruited through community screening programs. Individuals attending community screening programs will be approached by our study team (clinical research coordinators (CRC) and one of our Clinical Research Fellows) to provide information about the study and informed consent for participation. The team will emphasize that participation in the study is entirely voluntary, and declining participation will not affect the quality of healthcare they receive in any way. The risks (if any) and benefits of participation, as well as the study timeline and procedures, will be thoroughly explained. Written consent will be obtained from all participants.

### Data collection, examinations, and imaging

At baseline visit, whether in a community screening event or OCH, after gathering basic demographic data (age, gender, race), a questionnaire regarding medical history will be completed by the study team. The gathered data should include:

- Past ocular history (any eye diseases or ocular surgeries),
- Family history of retinal diseases (with specific focus on AMD),
- Past medical history
  - With the diagnosis date for each medical condition
- Past drug history,
  - steroid use (indication, route, dosage/frequency, start and end date),
- Social history
  - Smoking (pack/year)
  - work schedule (fixed hours or shift hours), working time and frequency,
  - Longest stretch of undisturbed sleep (<4 hours, 4–6 hours, 6–8 hours, > 8 hours)

Presenting distance visual acuity (VA) is measured monocularly using a modified Early Treatment of Diabetic Retinopathy Study (ETDRS) LogMAR-style chart at 4 meters, with the participant wearing their current habitual distance vision correction, if any. Pinhole VA will also be measured for each eye. Results will be documented in LogMAR.

At next step, Basic imaging protocol consisting of color fundus photography (CFP), optical coherence tomography (OCT) and optical coherence tomography angiography (OCTA) all using Topcon 3D OCT-1 (Maestro 2, Topcon Inc., Tokyo, Japan, wavelength 840nm, and speed of 50,000 A-scan per second) according to the settings mentioned in Table 1 would be conducted for both eyes of each participant without pupil dilation. Scans would be repeated in the case of motion artifacts and/or image quality value (IQV) < 30.

**Table 1:** Detailed settings of imaging in basic protocol.

Modalities	Imaging setting
Colour Fundus Photography (CFP)	Capture one image at 45 degrees, centred on the fovea (macula), with the optic disc clearly visible in the image
Optical Coherence Tomography (OCT)	Perform 3D volume 6x6 mm (resolution of 1024 and over scan count of 4), centred on the fovea (macula)
Optical Coherence Tomography Angiography (OCTA)	Perform en-face OCTA scans with 3x3mm, (scan resolution 320x320 and over scan count of 4), centred on the fovea (macula)

Images from the Basic Protocol will be graded by research fellows within 2 months for early signs of AMD. This is defined by:

1. Any presence of drusen, blood, atrophy, CNV, or pigment on CFP
2. Any changes in the outer retinal layers on OCT
3. Any flow signal on OCTA suggestive of a CNV

All subsequent follow-up visits will occur at SERI, and the follow-up plan will be determined based on the baseline visit imaging:

If none of the early signs of AMD are present at baseline, participants will go through the Basic Protocol in the annual follow up visits till the end of the study.

**Fig 1:**
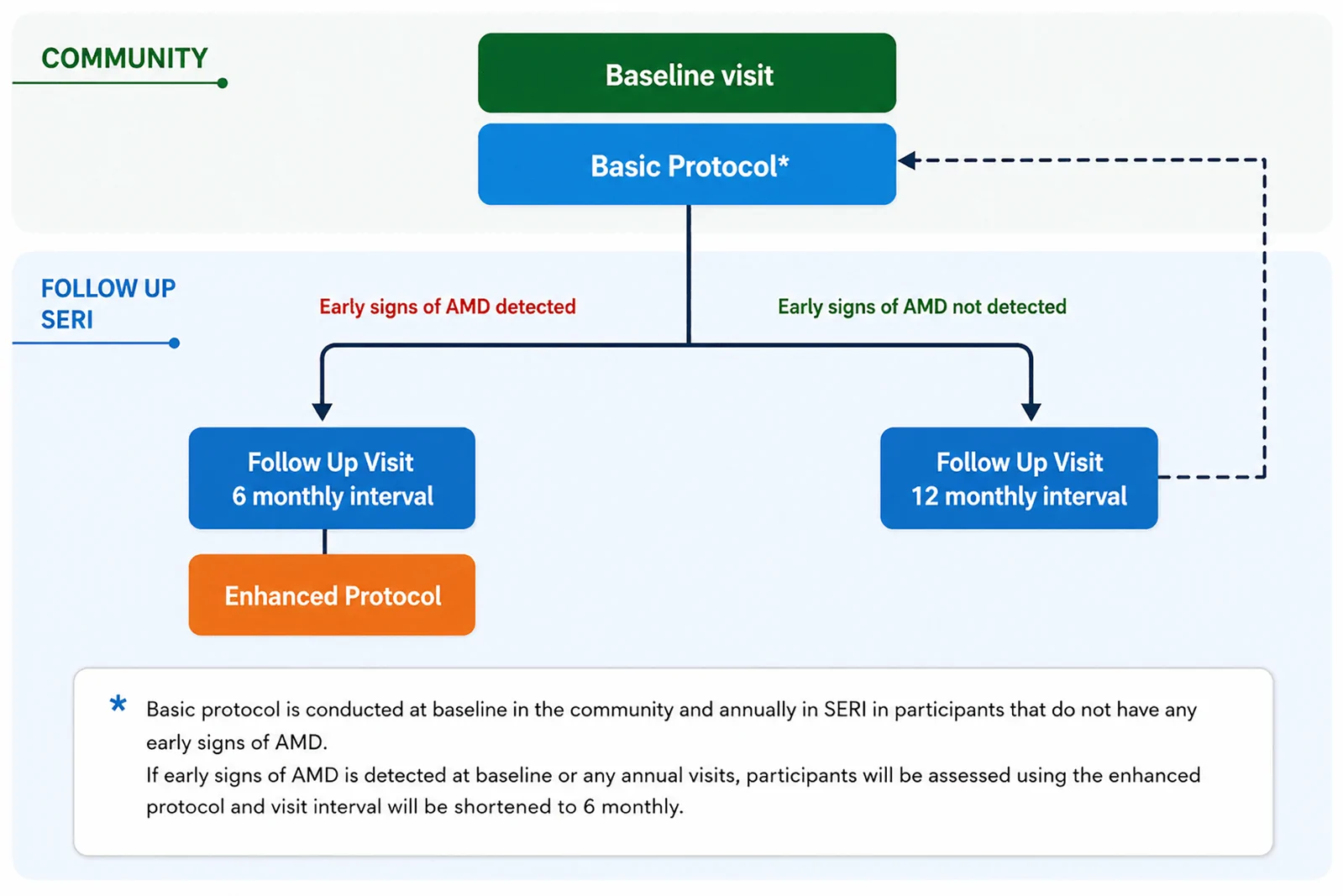
Disposition of participants.

If any of those signs are present at baseline or at any follow up visit, participants will be required to undergo the enhanced imaging protocol (Enhanced Protocol) during the 6 monthly follow up visits at SERI till the end of the 5-year follow-up. The participants’ flow and disposition are summarized in Fig 1.

At follow up visits, after reviewing the medical history records and measuring height, weight, and blood pressure, distance visual acuity with or without glasses and pinhole will be measured using the LogMAR chart for both eyes of each participant.

The Basic Protocol involves community-level imaging platforms, including CFP, OCT, and OCTA, which would be performed on both eyes of each participant (without pupil dilation) using Topcon 3D OCT-1 (Maestro 2) according to the settings mentioned in Table 1.

For participants who are part of the Enhanced Protocol, the imaging modalities will include Ultra Widefield Fundus Photography, OCT, FAF, and OCTA, and will all be performed according to the setting described in Table 2.

**Table 2:** Detailed enhanced imaging protocol.

Imaging modality	Imaging protocols (through dilated pupils)
Optical Coherence Tomography (OCT)	Heidelberg Spectralis (Heidelberg engineering, Heidelberg, Germany) Spectral-Domain OCT, wavelength of 870 nm, A-scan speed of 70,000 scans per second, axial resolution: 3.9 µm, transverse tissue resolution: 6 µm Perform cube scan 6x6mm scan, centred on the fovea (macula), 97 horizontal B-scan, 512 A-scans per line, Automatic Real Time (ART) 7–9, and Enhanced Depth Imaging (EDI) mode on.
Optical Coherence Tomography Angiography (OCTA)	Zeiss PlexElite (PlexElite 9000, Carl Zeiss Meditec) Swept-Source OCTA, wavelength of 1050nm, A-scan speed of 100,000 scans per second Perform en-face OCTA scans with 3x3mm and 6x6mm scan, centred on the fovea (macula) DREAM OCT (Vg200, Intalight) Swept-Source OCTA, wavelength of 1070nm, A-scan and speed of 400,000 A-scans per second Perform 29 x 24 mm OCTA scan (1536 x 1280, R2), centred on the fovea (macula)
Fundus Autofluorescence (FAF)	Heidelberg Spectralis OCT Excitation blue light of 488 nm and a barrier filter beginning at 500 nm Capture two images at 30 and 50 degrees (ART > 50), centred on the fovea (macula), with the optic disc clearly visible in the image Optos (Nikon, Tokyo, Japan) Ultrawide Field FAF
Ultrawide Field Fundus Photography	Optos (Nikon, Tokyo, Japan) Capture one shot at 200 degrees of the retina, centred on the fovea (macula) and includes optic disc

OCT scans will be repeated in the case of motion artifacts and/or low signal strength (SS) (Spectralis OCT Q < 30, Zeiss OCTA SS < 7). Automated retinal segmentation and slab boundaries in OCT and OCTA scans will be manually checked and corrected in case of segmentation errors.

Blood collection is optional and will only be performed for willing participants. Thirty milliliters of venous blood sample will be collected in EDTA tubes at any point during the 5-year follow-up period. The freshly collected blood has to be processed in the laboratory within 6 hours of collection. Upon Ficoll centrifugation of the blood specimen, a buffy coat layer containing mononuclear cells and neutrophils at the bottom of the tube will be isolated. These samples would be stored at –80°C for up to 15 years for genetic and metabolomic analysis at SERI.

### Grading of retinal imaging and outcomes to be measured

Multimodal imaging of the participants in the enhanced protocol will be graded monthly by research fellows according to Table 3.

**Table 3:** Detailed description of grading in the enhanced protocol.

Modality	Feature	Criteria
Ultrawide Field Fundus Photography	Drusen	The biggest drusen size within the 2-disc diameters of fovea • Small (≤63 µm) • Medium (>63 µm and ≤125 µm) • Large (>125 µm)
	AMD pigmentary abnormalities	Any definite hyper- or hypopigmentary abnormalities
	Hemorrhage^*^	Blood
	Fibrosis^*^	Obvious white or yellow mounds of fibrous-appearing tissue that were well defined in shape and appeared solid
	Atrophy^*^	Partial or complete depigmentation patches ≥175 μm in longest linear dimension that had ≥ 1 of these additional characteristics: sharply demarcated borders, visibility of underlying choroidal vessels, excavated or punched out appearance on stereoscopy of CFP
FAF		Atrophy:Hypoautofluorescent (black level similar to optic nerve head or retinal blood vessels) area with sharply demarcated borders FAF will also be used for confirming the presence of subretinal drusenoid deposits (areas of hyperfluorescent on the centre and surrounded by a Hyporeflective halo, forming a target appearance)
OCT	Central Subfield Thickness (CST)	The average thickness of the retina within the central 1-mm ETDRS subfield.
	Subfoveal choroidal thickness (SFCT)	The distance between the Bruch membrane and inner scleral surface measured in µm on the central B-scan using the caliper tool in the Heidelberg Spectralis platform.
	Drusen	• Soft drusen: focal thickening between the RPE and BM, ≥ 100μm diameter • Hard drusen: focal thickening between the RPE and BM, < 100μm diameter • Subretinal drusenoid deposits (SDD): mounds or cone-shaped structures with medium to high reflectivity, located either at the ellipsoid zone or between the ellipsoid zone and the RPE surface • Cuticular Drusen: blunted triangular elevations in a sawtooth configuration • Hyporeflective drusen core (HDC): drusen that have Hyporeflective space within them, with a minimum height of 40 μm • Pachydrusen: drusen located adjacent to pachyvessels
	Pigment epithelial detachment (PED)	>100μm separation of RPE and BM, • Serous PED: optically clear or hypo-reflective sub-RPE elevation • Drusenoid PED: dome-shaped RPE elevation with more homogeneous internal reflectivity, consistent with drusenoid material • Fibrovascular PED: irregular RPE elevation with heterogenous internal reflectivity
	Double layer sign (DLS)	a shallow irregular RPE elevation with separation between the RPE and Bruch’s membrane <100μm • Thin DLS: the space between RPE and Bruch’s membrane is occupied by a single zone of low-to-moderate reflectivity • Thick DLS: the space between RPE and Bruch’s membrane is occupied by at least two distinct zones with varying reflectivity Shallow irregular RPE elevation (SIRE): ≤ 100μm separation BM to RPE (height), ≥ 1000μm length of shallow continuous detachment, subset of DLS
	Intraretinal fluid (IRF) ^*^	Accumulation of the fluid in the retina, with formation of cystoid spaces.
	Subretinal fluid (SRF) ^*^	Accumulation of the fluid under the retina. Separation of the neurosensory retina from the RPE by fluid.
	Acquired vitelliform lesions (AVL)	Hyperreflective material between the RPE and photoreceptor layer, often appearing as a dome-shaped elevation with homogeneous hyperreflectivity of the subretinal deposit, although they may appear hyporeflective in some areas, particularly in the superior portions of the lesion.
	Hyperreflective material (HRM) ^*^	Exudation material that is hyperreflective, • subretinal, • sub-RPE space, • both-if RPE is not detectable HRM boundary • well-defined • ill-defined
	Intraretinal hyperreflective foci (HRF)	Hyperreflective lesions located within the neurosensory retinal layers (i.e., above the RPE band and exhibiting reflectivity comparable to that of the RPE band
	Irregular RPE	Nonspecific RPE changes, including molting of RPE, RPE thickening, hyperreflective spike of RPE et al.
	Choroidal hypertransmission	Increased signal transmission into the choroid beneath an area of RPE attenuation or loss
	Atrophy	including cRORA, iRORA (Incomplete RPE and outer retinal atrophy), cORA (Complete outer retinal atrophy), iORA (Incomplete outer retinal atrophy)
	Incomplete RPE and outer retinal atrophy (iRORA)	discontinuous signal hypertransmission into the choroid with attenuation or disruption of the RPE, and overlying photoreceptor degeneration
	Complete RPE and outer retinal atrophy (cRORA)	(1) a region of hypertransmission of at least 250 µm in diameter, (2) a zone of attenuation or disruption of the RPE of at least 250 µm in diameter, (3) evidence of overlying photoreceptor degeneration, and (4) absence of scrolled RPE or other signs of an RPE tear
OCTA		• Choroidal vascularity index (CVI) • Choriocapillaris flow area Those variables will be measured by the DREAM OCT in-built software and the average values at Early treatment diabetic retinopathy study (ETDRS) 0–1, 0–3, 0–6 mm areas will be documented. Abnormal flow signal in the outer retina/sub-RPE space or neovascular network visible on en-face view of those slabs

* These variables will be graded just in late AMD stage

The primary study outcome will be progression to late AMD, defined as 1- neovascular AMD (the development of macular neovascularization (MNV)) and/or 2- Geographic atrophy (GA, defined as atrophy lesions seen on CFP and FAF and further confirmed by the presence of complete retinal pigment epithelium and outer retinal atrophy (cRORA) on OCT, in the absence of present or previous MNV), during follow-up.

The rate of progression to late AMD will be reported, with event time assigned to the first visit at which GA or MNV is detected. The rate and time to event of progression from non-exudative neovascular AMD, detectable abnormal flow signal in the outer retina/sub-RPE space on OCTA and no exudative sign or macular fluid on OCT imaging, to exudative neovascular AMD will also be reported.

The prevalence of each multimodal imaging biomarkers alongside the rate of quantitative biomarkers and their longitudinal changes will be reported. By integrating those biomarkers with clinical (medical, drug and social history), genetic, and metabolomic data, we will assess their relative risk for progression and will present potential predictors. Furthermore, the identified prognostic factors may be used to explore clinically meaningful risk strata and inform the development of an AMD staging framework tailored to the Asian population. Using that classification, the rate of progression to different stages of AMD through years of follow-up will be reported.

### Statistical methods

Descriptive statistics will be used to summarize baseline demographic, systemic, ocular, and imaging characteristics of the study cohort. Continuous variables will be presented as mean ± standard deviation (SD) for normally distributed data, or median and interquartile range (IQR) for non-normally distributed data. Categorical variables will be summarized using frequencies and percentages. Normality will be assessed using histograms and the Shapiro–Wilk test.

All analyses will primarily be performed at the eye level. As both eyes from a participant may be included, inter-eye correlation will be accounted for using generalized estimating equations (GEE) or mixed-effects models where appropriate.

Because AMD progression will be assessed at scheduled study visits rather than continuously observed, the primary analysis will evaluate progression to late AMD over predefined follow-up intervals using discrete-time survival models. Standard Cox proportional hazards models may be performed as sensitivity analyses. Where appropriate, interval-censored survival models will be explored.

Variables considered for multivariable models may include demographic factors, systemic risk factors, smoking history, and multimodal imaging biomarkers, including drusen characteristics, pigmentary abnormalities, subretinal drusenoid deposits, hyperreflective foci, drusen volume, double-layer signs, OCTA flow abnormalities, and choroidal parameters. Hazard ratios (HR) and 95% confidence intervals (CI) will be reported.

Longitudinal continuous imaging parameters, including drusen volume, retinal thickness, and choroidal thickness, will be analyzed using linear mixed-effects models to account for repeated measurements over time and within-subject correlation. Logistic regression analyses may additionally be performed for binary outcomes such as conversion to late AMD within predefined follow-up intervals.

Where appropriate, exploratory multivariable models integrating these biomarkers may be constructed to investigate risk stratification. All statistical analyses will be performed using R software (R Foundation for Statistical Computing, Vienna, Austria) and/or IBM SPSS Statistics (IBM Corp., Armonk, NY, USA). All statistical tests will be two-sided, and a p-value <0.05 will be considered statistically significant.

### Safety measurements

The risks associated with participating in this study are minimal. Both eye examinations and blood collection are routine procedures that form part of standard medical care, posing no additional risk to participants.

While most eye examinations are non-invasive, minimal risks may arise during imaging procedures such as CFP, OCT, and OCTA, including mild discomfort due to light exposure during retinal imaging. Clinical examinations are part of the standard of care, with detailed instructions and comfortable environments to help alleviate any unease associated with these procedures.

Blood sample collection is also minimally invasive, with minor risks such as temporary pain, bruising, or infection at the venipuncture site. This collection will align with standard care practices.

Pupil dilation is a common ophthalmic procedure. While it is generally safe, there are potential risks and side effects that should be communicated to patients. There is a very small risk of angle closure glaucoma (an acute rise in eye pressure) from the administration of eye drops. This risk is estimated to be about 1 in 5,000 people. There is also a risk of a mild local allergic reaction to the eye drop. If participants develop these adverse effects (red painful eye, blurry vision, nausea and vomiting), they will be referred for immediate and appropriate treatment at Singapore National Eye Centre (SNEC).

### Data management and confidentiality of data and patient records

Research data will be coded, participants will be de-identified, and the master list will be encrypted with a password. Only delegated study team members/ personnel authorized by PI will be allowed access to patient data.

All collected samples will be processed in the laboratory in a de-identified manner to ensure participant confidentiality.

### Timeline of the study

The project received approval from the CIRB in October 2025. Up to the time of manuscript submission, two community screening events had been conducted (18/10/2025 and 06/06/2026), and a total of 64 participants had been recruited. We anticipate recruitment to be completed by Jun 2027, 5-year follow-up by August 2032, and grading and data analysis by the last quarter of 2032.

### Oversight, monitoring, and amendments

PRIME study is part of the Translational Asian Age-related Macular Degeneration Program (TAAP). This project has 4 different themes, which altogether are aimed at elucidating the mechanisms behind AMD in order to cultivate diagnostics, develop novel therapies and tools to better understand the impact of the disease from the patients’ perspective. TAAP projects are monitored by Scientific Advisory Board (SAB) annually.

All protocol amendments will be submitted by the principal investigator to the CIRB. Once approval is obtained, team members will be notified of the changes made, and the new protocol will be followed. All amendments will be included in the subsequent publications.

## Discussion

AMD is the most common cause of irreversible blindness in elderly in developed countries [11]. Given the chronic nature of the disease and the need for long-term management, AMD imposes a substantial burden on healthcare systems worldwide. Despite its clinical importance, there is a lack of data on the progression of AMD and optimal screening protocol. Identifying individuals at risk of progression to advanced AMD is essential for timely intervention.

Previous studies investigating AMD progression have been limited by retrospective design or relatively short follow-up periods [12–14]. Although several prospective longitudinal studies have recently been reported, those cohorts were predominantly composed of Caucasians and have been conducted on patients with clinically diagnosed AMD [15–18]. Consequently, the natural history and biomarkers of AMD progression in Asian populations remain incompletely understood.

The proposed approach marks the first large Asian AMD dataset aimed at understanding the natural progression of this disease. By integrating a comprehensive multimodal retinal imaging with clinical, genetic and metabolic data, this longitudinal cohort will contribute to the development of future screening and risk stratification frameworks.

### Strengths and limitations

The major strengths of this study include its prospective longitudinal design, five-year follow-up period, substantial sample size, and comprehensive multimodal phenotyping alongside clinical and genetic data.

Several limitations should also be acknowledged. First, pupil dilation will not be performed during the baseline community-based screening visit, which may reduce the gradability of some fundus photographs. Second, information on lifestyle and environmental factors, including dietary habits, physical activity, sunlight exposure, educational attainment, and socioeconomic status, will not be collected, limiting the ability to assess their contribution to disease progression. Finally, the number of participants who progress to late AMD during the study period may be limited, which could affect the statistical power of some analyses. Continued follow-up of this cohort beyond five years may help address this limitation and provide additional insights into the long-term natural history of AMD.

### Dissemination plans

Study results will be published in peer-reviewed academic journals and will be presented at national and international congresses.
